# Acute Cardiac Failure in a Pregnant Woman due to Thyrotoxic Crisis

**DOI:** 10.1155/2012/393580

**Published:** 2012-07-02

**Authors:** Nao Okuda, Mutsuo Onodera, Yumiko Tsunano, Emiko Nakataki, Jun Oto, Hideaki Imanaka, Masaji Nishimura

**Affiliations:** Emergency and Critical Care Medicine, Tokushima University Hospital, 3-18-15 Kuramoto, Tokushima 770-8503, Japan

## Abstract

*Introduction*. Cardiac failure during pregnancy is usually related to preeclampsia/eclampsia, rarely to hyperthyroidism. While hyperthyroidism can easily lead to hypertensive cardiac failure and may harm the fetus, it is sometimes difficult to distinguish hyperthyroidism from normal pregnancy. *Case Presentation*. We encountered a case of 41-year-old pregnant woman with hypertensive cardiac failure. Because we initially diagnosed as pre-eclampsia/eclampsia, Caesarian section was performed. However, her symptoms still persisted after delivery. After thyroid function test results taken on the day of admission were obtained on the fourth day, we could diagnose that her cardiac failure was caused by thyrotoxic crisis. *Conclusions*. Hypertensive cardiac failure due to hyperthyroidism during pregnancy is rare and difficult to diagnose because of similar presentation of normal pregnancy. However, physicians should be aware of the risks posed by hyperthyroidism during pregnancy.

## 1. Introduction

Cardiac failure during pregnancy is usually related to pre-eclampsia/eclampsia. While hyperthyroidism rarely causes cardiac failure in the parturient, it does weaken cardiac function and is harmful to the fetus. Early diagnosis and treatment are important to rescue both the mother and the fetus.

## 2. Case Presentation

**Figure 1:**
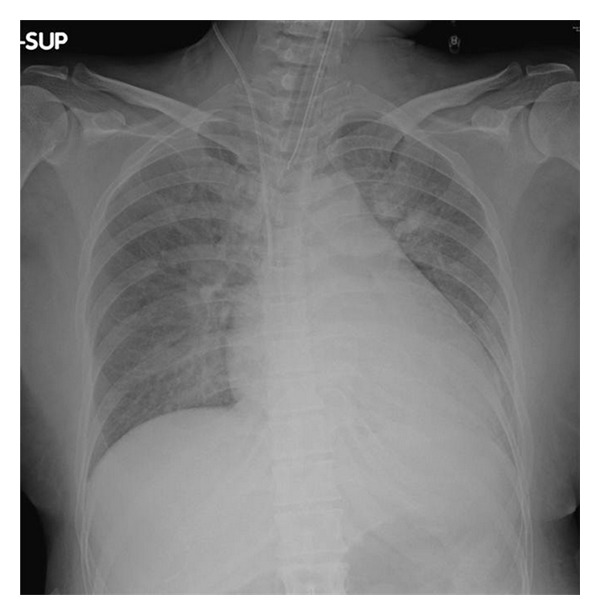
Chest X-ray on admission showing bilateral pulmonary infiltration and cardiac dilatation.

A 41-year-old pregnant woman—para III, gravida III—visited our hospital because of premature rupture of membrane at the 32nd week of gestation. She was 155 cm tall and weighed 49 kg. She had no appreciable past history before pregnancy. She was pointed out hypertension without proteinuria during pregnancy, but no medication had been prescribed. She was alert but showed severe hypertension and tachycardia. Her oxygen saturation rapidly deteriorated to 94% with reservoir mask supplying 10 L/min oxygen, she was intubated and transferred to our ICU. On admission, her blood pressure was 250/170 mmHg, pulse rate 180 beats/min, and body temperature 38.0°C. Laboratory data showed mild hepatic dysfunction and proteinuria. Chest X-ray exhibited bilateral pulmonary infiltration and cardiac dilatation (cardiothoracic ratio, 62%) (Figure 1). Echocardiography showed decreased wall motion (ejection fraction, 43%) and diffuse hypertrophy in the left ventricle.

She was initially treated as having acute cardiac failure due to pre-eclampsia/eclampsia. Nitroglycerin (0.5–1 *μ*g/kg/min), carperitide (0.05 *μ*g/kg/min), and furosemide (10 mg bolus) were administered to decrease preload and afterload, and Caesarian section was performed on the day of admission. Her baby was 1814 g female and APGAR score was 2/5. The baby was intubated and admitted to NICU because of general anesthesia. The baby grew uneventfully and discharged from hospital at 39 days old. On postoperative day (POD) 1, pulmonary congestion and oxygenation were improved, and she was successfully extubated. On POD 3, she was discharged to general ward; however, her tachycardia and hypertension still persisted.

On the same day, thyroid function tests taken on admission showed elevated serum-free triiodothyronine and thyroxine levels (7.0 pg/mL and 3.60 ng/dL, resp.) and declined thyroid-stimulating hormone level (<0.01 *μ*U/mL). Both antithyroglobulin antibody and antithyroid peroxidase antibody were positive (Table 1). We reviewed her history and physical findings and found that she had hoarseness and felt shortness of breath on effort before pregnancy, and her thyroid was diffusely enlarged. Grave's disease was diagnosed, and Thiamazole (60 mg) and propranolol hydrochloride (30 mg) were administered, whereupon her pulse rate declined from 100 to 80 beats/min. At the time of discharge, echocardiography showed improved wall motion (ejection fraction, 60%), and blood pressure was back to near normal. Thyroid function tests taken after discharge decreased serum-free triiodothyronine and thyroxine levels (6.3 pg/mL and 2.04 ng/dL, resp.) and declined thyroid-stimulating hormone level (<0.01 *μ*U/mL).

## 3. Discussion

We encountered a parturient who suffered acute hypertensive cardiac failure due to hyperthyroidism. There are several causes of hypertension during pregnancy (Table 2). Among these causes, pre-eclampsia/eclampsia is most common. Hyperthyroidism is less common cause for hypertension during pregnancy. However, approximately 10% of women with untreated thyrotoxicosis develop heart failure [1], and uncontrolled hyperthyroidism during pregnancy is associated with spontaneous abortion, premature labor, low birth weight, stillbirth, preeclampsia, and heart failure [2].

In this case, we initially assumed her symptoms were attributed to pre-eclampsia/eclampsia, simply because it is the most common cause of cardiac failure in pregnancy [3]. It is difficult to distinguish hyperthyroidism during normal pregnancy because some of the symptoms of pregnancy are similar to those of hyperthyroidism [4]. While she had several clues such as hoarseness and shortness of breath before pregnancy, we could not take her history because she was already intubated and sedated on ICU admission. However, careful examination of thyroid gland might lead to earlier diagnosis. Poor pregnancy weight gain and intrauterine growth retardation also corresponded to hypertension. We used vasodilators and diuretics to alleviate pulmonary congestion, which were also effective in thyrotoxicosis. Rapid delivery of the baby might reduce mother's oxygen demand; however, if hyperthyroidism was diagnosed earlier, Caesarian section might not be avoided.

In conclusion, we encountered a case of acute hypertensive cardiac failure secondary to undiagnosed hyperthyroidism during pregnancy. Although diagnosis of endocrine disorders during pregnancy is difficult, we should bear in mind that it may cause cardiac failure during pregnancy.

## Figures and Tables

**Table 1:** Blood analysis results for endocrine markers during hospitalization.

	Admission	Day 7	Reference range
FT3 (pg/mL)	7.0	17.5	0.93–1.75
FT4 (ng/dL)	3.6	6.8	2.3–3.7
TSH (*μ*U/mL)	<0.01	<0.01	0.65–5.55
TRAb (IU/L)		67.0	<4.5
ATA (IU/mL)		1393	<28
ATPA (IU/mL)		496.4	<16.0

FT3: free triiodothyronine, T4: free thyroxine, TSH: thyroid stimulating hormone, TRAb: TSH receptor antibody, ATA: antithyroglobulin antibody, ATPA: antithyroid peroxidase antibody.

**Table 2:** Causes of secondary hypertension during pregnancy.

Eclampsia/pre-eclampsia
Renovascular disease
Pheochromocytoma
Primary aldosteronism
Oral contraceptives
Sleep apnea syndrome
Hypothyroidism
Hyperthyroidism
Primary hyperparathyroidism
Cushing's syndrome

## References

[1] Sheffield JS, Cunningham FG (2004). Thyrotoxicosis and heart failure that complicate pregnancy. *American Journal of Obstetrics and Gynecology*.

[2] Mestman JH (1997). Hyperthyroidism in pregnancy. *Clinical Obstetrics and Gynecology*.

[3] Lin YS, Tang CH, Yang CY (2011). Effect of pre-eclampsia-eclampsia on major cardiovascular events among peripartum women in Taiwan. *American Journal of Cardiology*.

[4] Grigoriu C, Cezar C, Grigoras M (2008). Management of hyperthyroidism in pregnancy. *Journal of Medicine and Life*.

